# Efficacy and safety of same‐day discharge after atrial fibrillation ablation: A systematic review and meta‐analysis

**DOI:** 10.1002/clc.23778

**Published:** 2022-01-27

**Authors:** Sina Rashedi, Hamed Tavolinejad, Sina Kazemian, Mahta Mardani, Maryam Masoudi, Farzad Masoudkabir, Majid Haghjoo

**Affiliations:** ^1^ Department of Cardiac Electrophysiology, Tehran Heart Center Tehran University of Medical Sciences Tehran Iran; ^2^ Non‐Communicable Diseases Research Center, Endocrinology and Metabolism Population Sciences Institute Tehran University of Medical Sciences Tehran Iran; ^3^ Cardiac Primary Prevention Research Center, Cardiovascular Diseases Research Institute Tehran University of Medical Sciences Tehran Iran; ^4^ Department of Cardiac Electrophysiology, Rajaie Cardiovascular Medical and Research Center Iran University of Medical Sciences Tehran Iran; ^5^ Cardiac Electrophysiology Research Center, Rajaie Cardiovascular Medical and Research Center Iran University of Medical Sciences Tehran Iran

**Keywords:** atrial fibrillation, catheter ablation, efficacy, safety, same‐day discharge

## Abstract

Conventionally, patients have been admitted overnight after atrial fibrillation (AF) catheter ablation. Several centers have recently adopted a same‐day discharge (SDD) protocol for patients undergoing AF catheter ablation. We aimed to systematically review the current evidence for the safety and efficacy of SDD after AF catheter ablation. A systematic search was performed in PubMed, Embase, Scopus, Web of Science, and the Cochrane library until August 21, 2021. The risk of bias was assessed with the “Methodological Index for Non‐Randomized Studies” (MINORS). The pooled efficacy rate of SDD protocol (defined as the proportion of patients discharged the same day of ablation among the patients who were planned for SDD) was calculated. Meanwhile, pooled major complication rates and early readmission or emergency department (ED) visit rates were evaluated in successful and planned SDD groups separately. Overall, 12 observational studies consisting of 18,065 catheter ablations were included, among which 7320 (40.52%) were discharged the same‐day after ablation. The pooled efficacy was 90.3% (95% confidence interval [CI] [82.7–96.0]). The major complication rates were 1.1% (95%CI [0.5–1.9]), and 0.7% (95% CI [0.0–3.1]) in planned SDD and successful SDD groups, respectively. In addition, readmission/ED visit rate were 3.0% (95%CI [0.9–6.1]), and 3.1% (95% CI [0.8–6.5]) in the same groups. There were no significant differences between planned SDD and overnight groups with respect to major complication rate (risk ratio = 0.70, 95%CI [0.35–1.42], *p*‐value = .369). The available data indicates that SDD after AF ablation is safe and efficient. Further prospective and randomized studies are warranted to elucidate the safety of SDD after AF ablation and develop a standardized SDD protocol.

## 1 INTRODUCTION

During the last two decades and after the first report of catheter ablation as a treatment strategy for atrial fibrillation (AF), AF ablation has rapidly evolved into an advanced procedure.^1^ At present, catheter ablation of AF is frequently performed as a well‐established strategy in both paroxysmal and persistent AF to improve the quality of life, maintain sinus rhythm, and even reduce death or hospitalization in patients with AF and heart failure (HF).^2^, ^3^, ^4^ Contemporary catheter ablation technologies, improved techniques, and better experience have resulted in a peri‐procedural complication rate as low as 2.9%.^5^ Meanwhile, the rising incidence of AF^6^ paired with the expansion of indications for ablation has led to a near 10‐fold increase in the number of AF ablation cases.^7^, ^8^

Outpatient procedures are increasingly performed in cardiology with the advent of less invasive treatments. Same‐day discharge (SDD) has been studied and adopted as a safe and feasible approach after elective percutaneous coronary interventions,^9^ supraventricular tachycardia ablation,^10^ and implantation of cardiac electronic devices.^11^ Contrary to other ablation procedures, SDD after catheter ablation of AF seems more challenging due to a longer and more complex procedure that requires trans‐septal puncture, conscious sedation or general anesthesia, and intra‐procedural anticoagulation.^2^ Conventionally, patients have been admitted overnight following the ablation of AF. In recent years, several experienced centers have adopted an SDD protocol for selected patients undergoing AF catheter ablation^12^, ^13^, ^14^, ^15^, ^16^ to reduce costs, improve patient satisfaction, and increase resource efficiency. However, the data supporting this approach seems heterogenous, and AF ablation SDD has been largely based on the centers' clinical experience. To address this matter, we decided to systematically evaluate the current evidence for the safety and efficacy of SDD after AF catheter ablation in different practice settings and a broad range of patient characteristics.

## 2 METHODS

This systematic review and meta‐analysis were conducted in adherence with the Preferred Reporting Items for Systematic Reviews and Meta‐Analyses (PRISMA) statement.^17^ Since the Institutional Review Board (IRB) and ethical approvals were obtained for each of the included studies, no additional IRB or ethical approvals were required for this review. Details of the protocol for this review were registered on PROSPERO: International prospective register of systematic reviews (CRD42020222210).^18^

### 2.1 Search strategy

A systematic search using the keywords [“atrial fibrillation” OR “catheter ablation”] AND [“same‐day discharge” OR “day case”] was performed in PubMed, Embase, Scopus, Web of Science, and the Cochrane library from the date of database inception until August 21, 2021. No restrictions were applied regarding language or study type. The detailed search strategy in each electronic database is described in Supplementarry Online Material.

### 2.2 Study selection

After excluding duplicate results, all retrieved titles and abstracts were reviewed independently by two investigators (S.R. and M. Mar.) to select studies focusing on the SDD protocol after catheter ablation in patients with AF, using Rayyan application.^19^ According to the following eligibility criteria, the selected studies' full‐texts were independently reviewed for inclusion by three authors (S.R., H.T., M. Mar.). In case of discrepancy, agreement was achieved through discussion with another author (S.K.).

#### 2.2.1 Eligibility criteria

I. All original investigations (case–series, case–control studies, cohort studies, and clinical trials) in the adult population (aged ≥ 18 years) independent of sample size, or language, which included patients who underwent any type of AF catheter ablation and were discharged the same‐day without overnight hospital stay (SDD group),

AND

i. Reported the efficacy of SDD protocol (or data from which efficacy could be derived). Efficacy of SDD was defined as the proportion of patients discharged the same‐day, among patients who were initially planned for SDD.

OR

ii. Reported the safety outcomes defined as either ablation‐related major complications or readmission/emergency department (ED) visits in SDD group only or in comparison with patients who stayed at least one night in hospital after AF catheter ablation (overnight group).

The studies investigating the ablation of arrhythmias other than AF were excluded. Moreover, we excluded non‐original studies (reviews, editorials, letters, and meta‐analyses), as well as studies without a clear description of the outcomes. If studies were found with overlapping patient populations, only the one reporting the most comprehensive data was included in this review. Abstracts were excluded from the main results, but were used in a supplementary meta‐analysis.

### 2.3 Data extraction and outcome definition

The following information was extracted by two investigators (H.T. and S.R.) and double‐checked by a third (S.K.) to ensure accuracy: publication year, study design (prospective vs. retrospective), number of centers involved (single‐center vs. multicenter), total number of participants and ablations (as well as proportions of planned SDD, successful SSD, and overnight groups), mean age, male to female ratio, type of AF (paroxysmal or persistent), body mass index (BMI), left ventricular ejection fraction (LVEF), CHA_2_DS_2_VASc score, type of ablation, source of ablation energy, average procedure duration, discharge characteristics, length of follow‐up, SDD protocol, proportion of patients discharged the day of ablation among the patients who were planned for SDD (designated as efficacy rate), number of major ablation‐related complications (defined as vascular or hemorrhagic complications requiring intervention, pericardial tamponade or effusion requiring drainage, thromboembolic events [stroke/transient ischemic attack, acute coronary syndrome, air embolism, deep venous thrombosis/pulmonary thromboembolism], pulmonary edema, arrythmia requiring pacing, or mortality), and number of early readmissions or ED visits (from the day of discharge until 1–4 months).

For quantitative synthesis, we categorized study participants into three groups based on each discharge protocol and study methodology: (a) patients who received an indication for SDD (planned SDD), (b) patients among the first group who were successfully discharged the same day (successful SDD), and c) patients who were planned for overnight stay. Due to inherent differences between groups “a” and “b,” we pooled data separately for each group to minimize bias and achieve more methodologically robust results.

The outcomes of interest were SDD strategy efficacy, the rates of major complications, and early readmissions or ED visits in the SDD group. Comparing complications and readmissions between SDD and overnight groups is challenging, since lower‐risk patients would be selected for SDD, while high‐risk cases would be deemed unsuitable for discharge and admitted overnight. Moreover, the overnight group may also include patients who were at first planned for SDD but were admitted due to complications. Hence, we compared SDD versus overnight only in studies that satisfied the following conditions: (a) had a randomized design, OR reported data before and after SDD strategy was implemented in ablation center(s), OR compared between centers with different discharge protocols, and (b) reported data for planned SDD and planned overnight stay groups. This methodology was aimed to reduce the inherent selection bias that is expected when discharge decision is left to the discretion of physicians.

### 2.4 Quality assessment

The revised and validated version of “Methodological Index for Non‐Randomized Studies” (MINORS) was employed by two authors (M.M. and H.T.) independently.^20^ Discrepancies were resolved by discussion with a third author (S.R.). This index consists of 12 items (eight items for all non‐randomized studies and an additional four items for comparative studies), which are scored 0 (not reported), 1 (reported inadequately), or 2 (reported adequately). Therefore, the highest total scores for non‐comparative studies and comparative studies would be 16 and 24, respectively.

### 2.5 Statistical analysis

All analyses were conducted using Stata (version 16; Stata Corp) with a P‐value less than 0.05 indicating statistical significance. The pooled effect sizes of outcomes in the SDD group were calculated by Freeman‐Tukey double arcsine transformation using the “metaprop” command, a routine for pooling proportions.^21^ The pooled risk ratio (RR) and 95% confidence intervals (CI) of comparative outcomes between SDD and overnight groups were explored by the “metan” command.^22^ Random‐effect models were applied to calculate the pooled effect sizes due to presumed high heterogeneity among studies. The statistical heterogeneity was assessed by two tests: 1. The Cochrane's Q test with *p*‐value < .05 signifying heterogeneity,^23^ 2. The Higgin's I‐squared test.^24^

Sensitivity analyses were performed by excluding one study at a time from the analyses to assess each study's contribution to the pooled estimates. Publication bias was examined with the visual assessment of the funnel plots and statistical calculation of Egger's test with *p*‐value < .05, indicating the presence of publication bias.^25^

## 3 RESULTS

### 3.1 Search results and study characteristics

**Figure 1:**
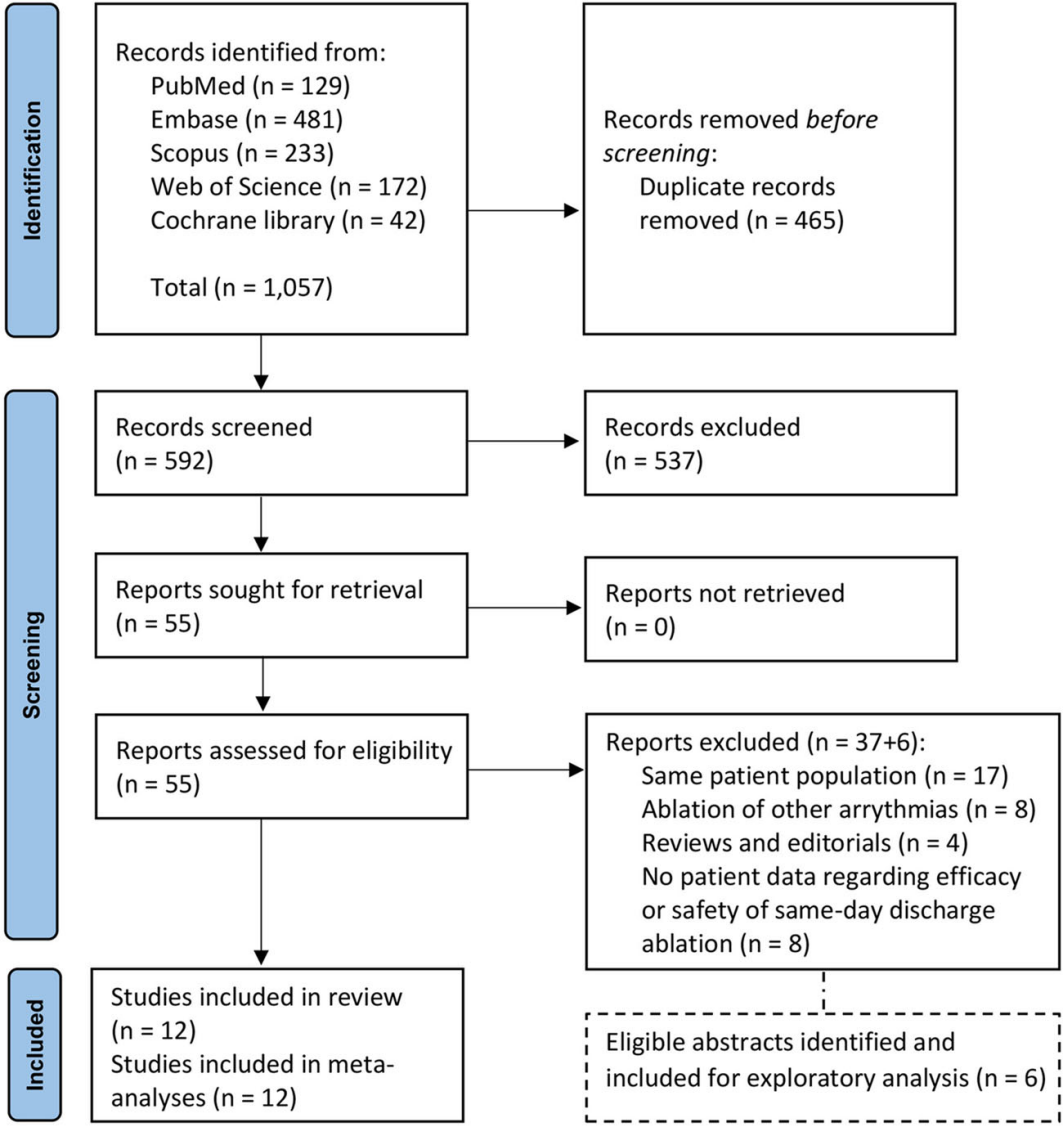
The PRISMA flow diagram

The systematic search of databases resulted in 1057 records. After excluding duplicates, 592 studies were screened, and 55 full texts were assessed for eligibility. The corresponding PRISMA flow chart is shown in Figure 1. Twelve observational studies were included.^12^, ^13^, ^14^, ^15^, ^16^, ^26^, ^27^, ^28^, ^29^, ^30^, ^31^, ^32^ Moreover, six abstracts were eligible for inclusion in this systematic review, but because they were not journal articles they were excluded and only considered in an exploratory analysis.^33^, ^34^, ^35^, ^36^, ^37^, ^38^

The studies were published from 2010 to 2021. The majority of studies were conducted in Europe (*n* = 5) and North America (*n* = 6). Regarding the study design, four studies were prospective,^14^, ^15^, ^26^, ^31^ and four involved more than one center.^16^, ^28^, ^30^, ^32^ In total, these studies included 18,065 patients, among which 7320 (40.52%) were discharged the same day. Mean age was between 56.0 and 66.6 years, and 67.08% were males. The type of AF (as reported in nine studies) was paroxysmal and persistent in 60.98% and 39.02%, respectively. The follow‐up duration ranged from three days to twelve months. The primary ablation method was pulmonary vein isolation (PVI) utilizing radiofrequency or cryoballoon with the mean procedure duration ranging from 63.5–201 min. The characteristics of the included studies are presented in Table 1. The abstracts are summarized in Table S1. Table S2 demonstrates the procedural and discharge protocol characteristics of each study.

**Table 1:** Characteristics of the included studies

Study, year	Country	Study design	Number of participants (SDD/ON)	Mean age (years)	Male/Female	Type of AF (paroxysmal/persistent)	Patients with heart failure/LVEF (%)	Body mass index (kg/m2)	CHA2DS2VASc score	Follow‐up duration (months)	MINORS score
Haegeli et al., 2010^15^	Canada	Prospective, Single‐center	206 patients	56.0 ± 9.3	152/54	171/35	NR/59.2 ± 4.7	NR	NR	NR	12/16
			230 ablations (205/25)								
Ignacio et al., 2018^26^	Argentina	Prospective, Single‐center	195	S‐SDD:	152/43	154/41	NR/	S‐SDD:	CHADS2:	1	20/24
				57 (49‐66)							
			(58/137)	ON: 62 (52–66)			S‐SDD: 60 (59‐64)	28 (26–31)	0 → 61 (31.3%)		
								ON: 27 (25–31)	1 → 62 (31.8%)		
							ON: 60 (50‐62)		2 → 43 (22.1%)		
									≥3 → 29 (14.9%)		
Opel et al., 2019^14^	UK	Prospective, Single‐center	276 (272/4)	61 ± 0.7	169/107	218/58	NR/NR	NR	CHA_2_DS_2_VASc	3	12/16
									0 → 72 (26%)		
									1 → 55 (20%)		
									2 → 72 (26%)		
									≥3 → 77 (28%)		
Bartoletti et al., 2019^13^	UK	Retrospective, Single‐center	785 (143/642)	59 ± 11.0	535/250	S‐SDD: 108/35	NR/NR	S‐SDD: 29.5 ± 5.2	CHA_2_DS_2_VASc:	NR	15/24
									S‐SDD: 2 ± 1		
								ON: 29.4 ± 5.0	ON: 1 ± 1		

Akula et al., 2020^27^	USA	Retrospective, Single center	571 (426/145)	61.7 ± NR	384/187	350/221	NR/	NR	NR	1	17/24
							P‐SDD: 56.4 ± NR				
							ON: 59.2 ± NR				
Creta et al., 2020^16^	UK and Italy	Retrospective, Multicenter	2628 (727/1901)	62.4 ± 11.6	1830/798	1350/1278	Cardiomyopathy:	NR	NR	3	18/24
							P‐SDD:				
							79 (10.9%)				
							ON:295 (15.5%)/				
							NR				
Deyell et al., 2020^28^	Canada	Retrospective, Multicenter	3054 (2418/636)	60.4 ± 9.5	2224/830	1907/1147	Heart failure: 402 (13.2%)/	NR	NR	1	17/24
							NR				
He et al., 2020^29^	UK	Retrospective, Single‐center	951	60.9 ± 11.6	395/572	620/330	Cardiomyopathy:	NR	NR	4	18/24
			(407/544)				P‐SDD: 35 (9%)				
							ON: 23 (4%)/				
							NR				
Kowalski et al., 2020^30^	USA	Retrospective, Multicenter	2374 (1194/1180)	64.9 ± 10.5	1618/	NR	S‐SDD: 119 (10%)	S‐SDD: 30 ± 6	CHA_2_DS_2_VASc:	1	18/24
					756		ON: 191 (16%)/		S‐SDD: 1.4 ± 1.0		
							S‐SDD: 56 ± 9	ON: 31 ± 6	ON: 2.2 ± 1.4		
							ON: 55 ± 10				
Reddy et al., 2020^12^	UK	Retrospective, Single‐center	448 patients	60.3 ± 9.9	326/126	269/138	NR/NR	NR	NR	6	20/24
			452 ablations (168/284)								
Rajendra et al., 2020^31^	USA	Prospective, Single‐center	82 ablations	59.2 ± 11.37	50/32	82/0	S‐SDD: 2 (4.9%)	29.9 ± 4.9	CHA_2_DS_2_VASc:	3	21/24
			41/41				ON: 0 (0%)		0: 16/1: 24/2: 24/3: 16/4: 2		
Field et al., 2021^32^	USA	Retrospective, Multicenter	6600 ablations	66.6 ± NR	4365/2235	NR	NR	NR	CHA_2_DS_2_VASc:	12	20/24
			1660/4940						SDD: 3.01 ± 1.71		
									ON: 3.01 ± 1.73		

*Note*: Data are reported as number (percentage), mean ± standard deviation, or median (interquartile range) in case of non‐normal distribution.

Abbreviations: AF, atrial fibrillation; CFAE, complex fractionated atrial electrograms; MINORS, methodological index for non‐randomized studies; LVEF, left ventricular ejection fraction; NR, not reported; ON, overnight; P‐SDD, planned same‐day discharge; S‐SDD, successful same‐day discharge.

Regarding the quality of included studies, two were non‐comparative, which scored between 12 out of 16. The remaining ten comparative studies scored 15–21 from a total of 24 (Table 1). Figure S1 demonstrates the quality assessment of the included studies through MINORS criteria.

### 3.2 Efficacy

**Figure 2:**
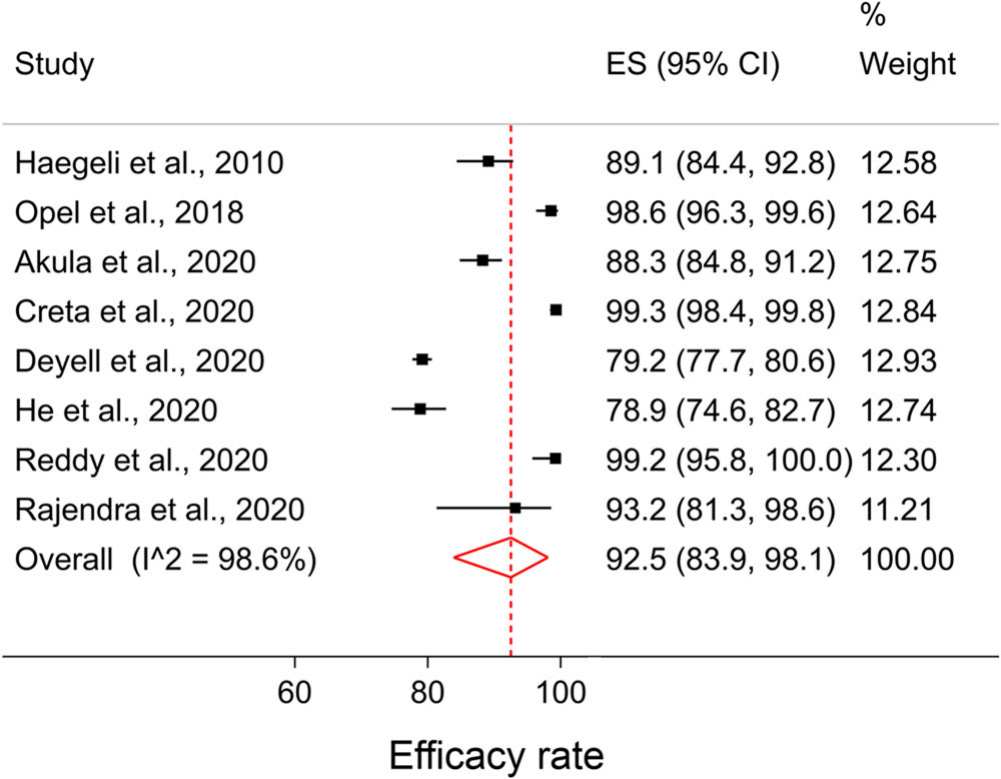
Forest plot representing the efficacy rate of same‐day discharge. CI, confidence interval; ES, effect size (percentage)

The SDD protocol efficacy was reported in eight full‐text articles (*n* = 8071). The pooled efficacy rate was 92.5% (95% confidence intereval [CI]: [83.9–98.1]; Figure 2). The overall heterogeneity was high (I‐squared = 98.6% and *p*‐value < 0.001). The main reasons for failure to discharge were ablation‐related complications,^14^, ^15^, ^27^, ^28^, ^31^, ^34^, ^35^, ^36^ non‐ablation‐related medical care,^27^, ^28^ late completion of ablation,^12^, ^15^, ^28^, ^35^ and patient preference.^27^, ^31^

In an exploratory analysis, four abstracts including efficacy rate were also considered (*n* = 1233). After adding abstracts, the pooled efficacy indicated that 90.3% of all planned SDD cases were successfully discharged (95% CI: [82.7–96.0]; I‐squared = 98.4% and *p*‐value < .001; Figure S2).

### 3.3 Major complications

**Figure 3:**
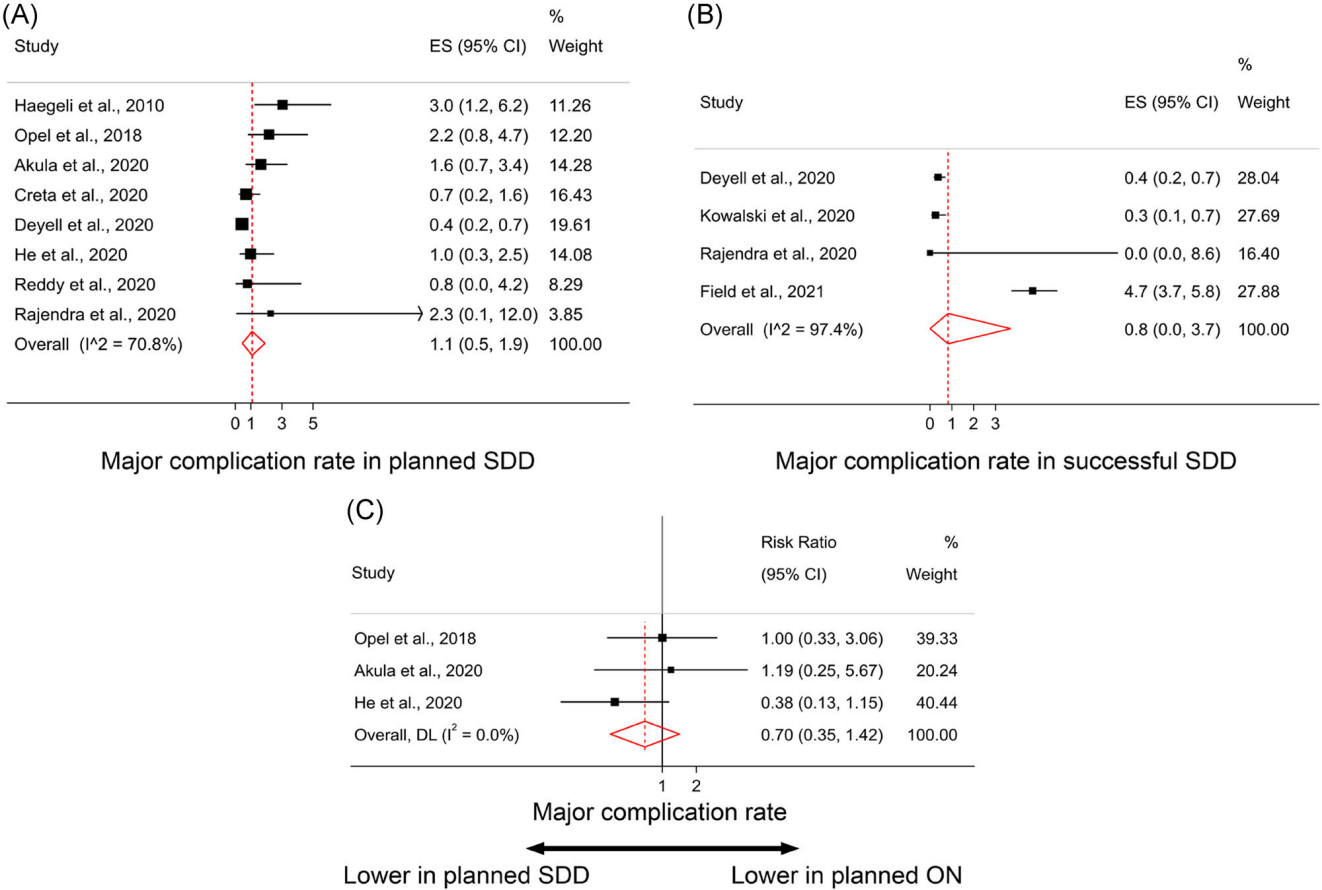
Pooled proportion of major complications in (A) planned same‐day discharge (SDD) group, and (B) Successful SDD group; (C) Comparison of major complication rates between planned SDD and overnight admissions. CI, confidence interval; ES, effect size (percentage)

The major complication rate in patients with planned SDD, based on eight full‐text studies (*n *= 5293) was 1.1% (95% CI: [0.5–1.9]; Figure 3) with substantial heterogeneity (I‐squared = 70.8% and *p*‐value < .001). Moreover, major complication rate in patients with successful SDD, based on four full‐text studies (*n* = 5238) was 0.8% (95% CI: [0.0–3.7]; I‐squared = 97.4% and *p*‐value < .001; Figure 3).

We also evaluated major complications in both planned and successful SDD groups with abstracts. The major complication rate in planned SDD groups, after including two abstracts (*n* = 231) was 1.1% (95% CI: [0.5–1.9]; I‐squared = 67.1% & *p*‐value < .001). The major complication rate in successful SDD group, after adding three abstracts (*n* = 235) was 0.7% (95% CI: [0.0–3.1]; I‐squared = 95.1% and *p*‐value < .001; Figure S3), similar compared to full‐text studies.

Based on our study protocol, a comparison of major complications in planned SDD versus planned overnight stay groups was possible in three studies (*n* = 1693), two of which compared time periods before and after implementation of SDD protocol,^27^, ^29^ and one compared two centers with and without SDD protocol.^14^ The pooled risk ratio for planned SDD compared to planned overnight stay was 0.70 (95% CI: [0.35–1.42]; I‐squared = 0.0% and *p*‐value = .369; Figure 3C).

### 3.4 Readmission or ED visits

**Figure 4:**
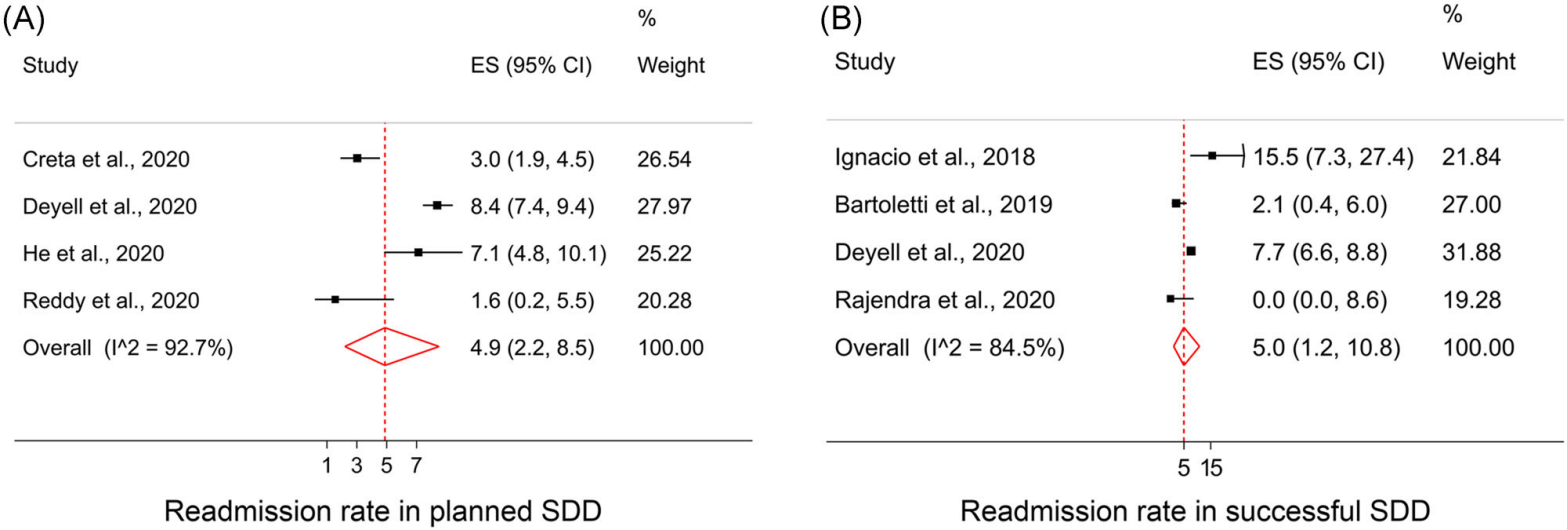
Pooled proportion of early readmissions in (A) planned same‐day discharge (SDD) group, and (B) successful SDD group. CI, confidence interval; ES, effect size (percentage)

The pooled early readmission or ED visit rate of four full‐text investigations in patients with planned SDD strategy (*n* = 4317) yielded a rate of 4.9% (95% CI [2.2–8.5]; I‐squared = 92.7% and *p*‐value < .001; Figure 4). In addition, the pooled early readmission or ED visit rate in patients with successful SDD strategy, based on four full‐text studies (*n* = 3302) was 5.0% (95% CI: [1.2–10.8]; I‐squared = 84.5% and *p*‐value < .001; Figure 4).

We observed similar early readmission or ED visit rate in both SDD groups with consideration of full‐texts and related abstracts. The early readmission or ED visit rate in planned SDD groups, after entering two abstracts (*n* = 231) was 3.0% (95% CI: [0.9–6.1]; I‐squared = 93.0% and *p*‐value < .001). The rate of early readmission or ED visit in successful SDD strategy, including three abstracts (*n* = 652) was 3.1% (95% CI: [0.8–6.5]; I‐squared = 87.1% and *p*‐value < .001; Figure S4).

Comparison of early readmission or ED visit rate was only available from one study by He et al.^29^ This study found that after four months of follow‐up readmissions occurred in 7.0% of the SDD groups versus 5.8% of the overnight admission group.

### 3.5 Sensitivity analysis and publication bias

According to sensitivity analyses, the removal of no single study changed the effect sizes or heterogeneity statistics significantly (Table S3). No significant publication bias (based on Egger's test) was noted in pooled total efficacy rate (*p*‐value = .104), major complication rate in successful SDD (*p*‐value = .975), and readmission rates in planned (*p*‐value = .256) and successful SDD (*p*‐value = .733); however, major complication rates in planned SDD indicated the presence of publication bias (*p*‐value = .030). The funnel plots are represented in Figure S5.

### 3.6 Cost analysis

Five studies investigated the economic impact of SDD protocol by subtracting the costs related to readmissions/presentation to ED from cost savings because of early discharge.^12^, ^16^, ^26^, ^29^, ^30^ All the investigations concluded that this strategy could lead to considerable cost‐saving, as well as more hospital facilities available for the rising number of patients awaiting AF catheter ablation. Due to considerable differences regarding admission and medical care costs among the centers, conducting a meta‐analysis was not feasible.

## 4 DISCUSSION

In this systematic review and meta‐analysis, we comprehensively evaluated the available evidence for the efficacy and safety of SDD after AF catheter ablation. The results indicate an acceptable efficacy or success rate of 92.5%, low rates of 1.1%, and 0.8% major peri‐procedural complications, and 4.9% and 5.0% readmission rates in planned and successful SDD, respectively. Moreover, there was no statistical difference in complications between SDD and overnight groups. Notably, the rate of major complications was marginally lower in successful SDD compared to planned SDD. This is expected because patients in the planned SDD groups may develop complications and stay overnight (failed SDD patients).

It should be noted that there was no evidence from randomized clinical trials on the safety and feasibility of SDD after AF catheter ablation, as all included studies had an observational design. Although we aimed to minimize selection bias by following a strict methodology, it should be noted that several characteristics could have affected the decision for discharge or admission, especially but not only in studies that consider successful SDD cases. There is currently only one ongoing prospective single‐center, single‐arm trial with approximately 50 participants assigned to SDD after AF catheter ablation to evaluate the rate of successful SDD with three months of follow‐up (NCT04199702).^39^ The strategy of SDD after AF catheter ablation clearly needs further investigation. There are currently no consensus about the protocol of SDD after AF ablation, and the most recent 2020 European Society of Cardiology AF practice guidelines does not provide any recommendations about early discharge after this procedure.^7^ Notably, at the time of the current COVID‐19 pandemic, SDD is of even greater importance to improve patient and personnel safety. In this context, this meta‐analysis could help cardiac electrophysiologists to reach a conclusion regarding the current evidence and aid in decision making at the individual patient level as well as developing hospital policies. Combining the results of studies from different cardiovascular centers with nonuniform protocols gives a more accurate estimate of the feasibility of SDD. Furthermore, pooling the available data can more reliably determine the incidence of rare complications.

Previously, Sahashi et al. published a similar systematic review^40^; however, they did not address the success rate of SDD in their study and did not attempt to consider overnight admissions as a control group. This problem was addressed in a later meta‐analysis by Prasitlumkum et al.,^41^ who attempted to compare SDD and overnight groups in their study, but as mentioned earlier, this comparison is prone to significant selection bias. This is because high‐risk cases will be admitted and it is patients with lower‐risk who are selected for SDD, which will result in no difference in complications or even lower complications in the SDD group. Moreover, the planned SDD and successful SDD groups should be pooled separately, since an analysis of each group conveys a different message. Planned SDD indicates the efficacy and safety of SDD as a treatment approach and estimates the rates if patients receive SDD indication based on the protocols of included studies (Table S2). On the other hand, the successful SDD group focuses on patients with the lowest risk. Finally, we have pooled the SDD efficacy rate, which indicates that among all AF ablation cases, a very high proportion can be successfully discharged the same day of the procedure.

Three studies had a larger number of cryoballoon than radiofrequency ablations among SDD cases.^12^, ^16^, ^29^ These observations may signal a preference for cryoballoon ablation cases to be selected for SDD, since patients who undergo repeat procedures and those who require additional and more complex lesions are more likely to receive radiofrequency ablation. In other words, it might be that radiofrequency ablation cases had a higher risk due to comorbidities and were more likely to be admitted overnight. Other procedural factors that may be associated with complications and affect discharge decision include procedure duration, radiofrequency delivery/freezing times, and performing additional ablations beyond PVI alone. However, we could not derive a meaningful conclusion regarding these potential associations due to the heterogeneity of reporting. Previously, two large cohorts and a meta‐analysis failed to show a procedure‐related predictor of complications, although there was a trend towards higher complications rates with longer procedure durations and complex fractionated atrial electrogram (CFAE) ablations.^5^, ^42^, ^43^

While SDD appears to be a convenient option after AF catheter ablation, some critical questions remain to be answered. First, there is hardly any data indicating which patients are appropriate candidates for SDD. Essentially, most studies involved in this review included all consecutive cases undergoing AF ablation without a decisive exclusion criterion and left the decision to admit or discharge to the treating physicians. Notably, most studies predominantly involved younger patients with mean ages close to 60 years (Table 1); thus, these findings should not be extrapolated to older and frailer patients. Kowalski et al. reported younger age, lower body mass index, lower CHA_2_DS_2_VASc score, and the absence of heart failure to be more frequent in the SDD group.^30^

Although some studies describe a pre‐specified protocol for AF ablation SDD adopted in their centers, these protocols are heterogeneous (Table S2) as there is currently no consensus about a safe and feasible approach. Transthoracic echocardiography was mentioned as part of the postablation protocol,^14^, ^16^, ^30^ although, at some centers, it was not required in the absence of clinical suspicion for pericardial effusion.^12^, ^13^, ^15^, ^28^ In most centers, pre‐procedure transesophageal echocardiograms to identify clots were reserved for patients with suboptimal anticoagulation before ablation,^12^, ^13^, ^15^, ^16^, ^28^, ^29^ whereas one study reported routine transesophageal echocardiography before each case.^30^ Management of anticoagulation is an essential consideration before, during, and after ablation, but the optimal approach for peri‐procedural anticoagulation is the subject of ongoing research.^44^, ^45^ Importantly, some centers have specified their anticoagulation strategy for implementing SDD.^16^, ^28^, ^29^, ^30^ To achieve hemostasis, a number of centers routinely use protamine sulfate,^14^, ^16^ femoral compression devices,^14^ and Z‐sutures.^16^, ^30^ Other shared features of SDD protocols include a period of observation before discharge, early ambulation, a watchful follow‐up program by clinic visits or phone calls, and patient education.^12^, ^13^, ^16^, ^27^, ^28^, ^30^ Moreover, studies report the proximity and ease of access to the hospital as a pre‐requisite for SDD.^16^, ^28^ AF ablation SDD safety could be further improved by incorporating novel strategies to minimize complications.^46^, ^47^ In conclusion, based on the available evidence, SDD after AF ablation seems to be best performed when cardiovascular centers adopt a pre‐specified and patient‐centered protocol.

An important aspect regarding the safety and feasibility of SDD is experience. The included studies mainly come from expert high‐volume referral hospitals. It has been demonstrated that a low volume center is associated with less favorable outcomes and a higher risk of postprocedural mortality^48^; therefore, these findings cannot be generalized to all AF ablation settings. On another note, while an increased cost‐efficiency with SDD seems plausible,^12^, ^16^, ^29^, ^30^ more data is needed before conclusions can be drawn. Importantly, patient preference should be considered in the decision to discharge. In patients undergoing elective coronary stenting and cardiac electronic device implantations, studies have indicated a higher patient satisfaction with SDD^9^, ^49^, ^50^; however, there is currently no data for SDD to improve patient experience after AF ablation, and this is an area for future research.

Over one‐third of patients with new‐onset AF have a history of HF, while more than half the patients with incident HF have a history of AF.^51^ During the past decade, strong evidence has been accumulated, showing better outcomes associated with AF catheter ablation in the presence of heart failure with reduced ejection fraction (HFrEF).^2^, ^4^ These facts denote an expected increase in the number of patients with concomitant AF and HFrEF occupying the beds of AF ablation facilities. None of the studies included in this analysis systematically evaluated SDD for patients with AF and HFrEF. Since patients with HFrEF have more comorbidities and are at a higher risk for unfavorable outcomes, the safety and feasibility of SDD for this subgroup needs to be evaluated in future investigations.

### 4.1 Study limitations

The absence of eligible randomized controlled trials in the literature limits the reliability of these findings. We found that characteristics such as procedure duration are not balanced between SDD and overnight groups in some included articles (Table S2). This limitation added to the lack of consensus or pre‐determined criteria for SDD, and heterogeneity in SDD protocols should be considered in interpreting the results and signifies the need for randomized studies to draw robust conclusions. Moreover, six studies did not include overnight cases as a control group, and after the quality assessment, some articles were found to have suboptimal methodologies. There was substantial heterogeneity in the outcomes. However, after extensive workup with subgroups and sensitivity analyses, we did not find a source of statistical heterogeneity. The included studies were published between 2010 and 2021, and due to the rapid evolution of mapping and ablation technologies, caution is advised for expanding these estimates to current practice. Since patient‐level data were not available, the complications included in this review are limited to those reported in the original papers. Furthermore, some of these complications may not have occurred immediately after discharge, and it is not clear whether they would have been prevented by inpatient monitoring.

## 5 CONCLUSION

The available data from non‐randomized observational studies indicate that SDD after catheter ablation of AF is a safe and feasible approach. The incidence of major complications and the rates of readmission/ED visits are reasonably low and comparable to patients who are admitted overnight. Prospective and randomized studies are warranted to further elucidate the safety of SDD after AF ablation, identify appropriate candidates, and develop a standardized SDD protocol.

## CONFLICT OF INTERESTS

The authors declare that there are no conflict of interests.

## Supporting information

Supporting information.

## Data Availability

Data are available upon a reasonable request to the corresponding author.
