# Supplementary material for: Efficacy and safety of same‐day discharge after atrial fibrillation ablation: A systematic review and meta‐analysis
Source: Clin Cardiol. 2022 Jan 27;45(2):162–72. doi: 10.1002/clc.23778 (PMC8860483; doi:10.1002/clc.23778)
Supplement: Supplementary file 1 — Supporting information. [file CLC-45-162-s001.docx]

Efficacy and safety of same-day discharge after atrial fibrillation ablation: a systematic review and meta-analysis

Supplementary Materials

[**Supplemental methods 2**](#_Toc85684714)

[**Search strategy 2**](#_Toc85684715)

[**Supplementary Figures S1-S5 7**](#_Toc85684716)

[**Supplementary Figure S1 8**](#_Toc85684717)

[**Supplementary Figure S2 9**](#_Toc85684718)

[**Supplementary Figure S3 10**](#_Toc85684719)

[**Supplementary Figure S4 11**](#_Toc85684720)

[**Supplementary Figure S5 12**](#_Toc85684721)

[**Supplementary Table S1 13**](#_Toc85684722)

[**Supplementary Table S2 14**](#_Toc85684723)

[**Supplementary Table S3 18**](#_Toc85684724)

**Supplemental methods**

Search strategy

## PubMed (August 21, 2021)

| **Search** | **Query** | **Results (No.)** |
| --- | --- | --- |
| #1 | "Atrial fibrillation*"[MeSH] OR "Catheter Ablation"[Mesh] OR "Auricular fibrillation*"[All Fields] OR "Transvenous Catheter Ablation*"[All Fields] OR "Electrical Catheter Ablation*"[All Fields] OR "Electric Catheter Ablation*"[All Fields] OR "Transvenous Electric Ablation*"[All Fields] OR "Transvenous Electrical Ablation*"[All Fields] OR "Radiofrequency Catheter Ablation*"[All Fields] OR "Percutaneous Catheter Ablation*"[All Fields] OR "atrial fibrillation catheter ablation*"[All Fields] OR "atrial fibrillation ablation*"[All Fields] OR "AF ablation*"[All Fields] OR "AF catheter ablation"[All Fields] OR "pulmonary vein isolation"[All Fields] OR "RF catheter ablation"[All Fields] OR "cryothermal ablation"[All Fields] OR "cryoballoon ablation"[All Fields] OR "cryoablation"[All Fields] | 90,336 |
| #2 | "same-day"[All Fields] OR "same day"[All Fields] OR "same-day discharge"[All Fields] OR "same day discharge"[All Fields] OR "day case"[All Fields] OR "day-case"[All Fields] OR "day stay"[All Fields] | 19,786 |
| #3 | #1 AND #2 | 129 |

## Embase (August 30, 2021)

| **Search** | **Query** | **Results (No.)** |
| --- | --- | --- |
| #1 | "Atrial fibrillation*"/mj OR "Catheter Ablation"/mj OR "Auricular fibrillation*":ti,ab,kw OR "Transvenous Catheter Ablation*":ti,ab,kw OR "Electrical Catheter Ablation*":ti,ab,kw OR "Electric Catheter Ablation*":ti,ab,kw OR "Transvenous Electric Ablation*":ti,ab,kw OR "Transvenous Electrical Ablation*":ti,ab,kw OR "Radiofrequency Catheter Ablation*":ti,ab,kw OR "Percutaneous Catheter Ablation*":ti,ab,kw OR "atrial fibrillation catheter ablation*":ti,ab,kw OR "atrial fibrillation ablation*":ti,ab,kw OR "AF ablation*":ti,ab,kw OR "AF catheter ablation":ti,ab,kw OR "pulmonary vein isolation":ti,ab,kw OR "RF catheter ablation":ti,ab,kw OR "cryothermal ablation":ti,ab,kw OR "cryoballoon ablation":ti,ab,kw OR "cryoablation":ti,ab,kw | 217,769 |
| #2 | "same-day":ti,ab,kw OR "same day":ti,ab,kw OR "same-day discharge":ti,ab,kw OR "same day discharge":ti,ab,kw OR "day case":ti,ab,kw OR "day-case":ti,ab,kw | 33,473 |
| #3 | #1 AND #2 | 481 |

## Scopus (August 21, 2021)

| **Search** | **Query** | **Results (No.)** |
| --- | --- | --- |
| #1 | (( TITLE-ABS-KEY  ("Atrial fibrillation*" OR "Catheter Ablation" OR "Auricular fibrillation*" OR "Transvenous Catheter Ablation*" OR "Electrical Catheter Ablation*" OR "Electric Catheter Ablation*" OR "Transvenous Electric Ablation*" OR "Transvenous Electrical Ablation*" OR "Radiofrequency Catheter Ablation*" OR "Percutaneous Catheter Ablation*" OR "atrial fibrillation catheter ablation*" OR "atrial fibrillation ablation*" OR "AF ablation*" OR "AF catheter ablation" OR "pulmonary vein isolation" OR "RF catheter ablation" OR "cryothermal ablation" OR "cryoballoon ablation" OR "cryoablation")) AND ( TITLE-ABS-KEY ("same-day" OR "same day" OR "same-day discharge" OR "same day discharge" OR "day case" OR "day-case"))) | 233 |

## Web of Science (August 21, 2021)

| **Search** | **Query** | **Results (No.)** |
| --- | --- | --- |
| #1 | ALL=(Atrial fibrillation* OR Catheter Ablation OR Auricular fibrillation* OR Transvenous Catheter Ablation* OR Electrical Catheter Ablation* OR Electric Catheter Ablation* OR Transvenous Electric Ablation* OR Transvenous Electrical Ablation* OR Radiofrequency Catheter Ablation* OR Percutaneous Catheter Ablation* OR atrial fibrillation catheter ablation* OR atrial fibrillation ablation* OR AF ablation* OR AF catheter ablation OR pulmonary vein isolation OR RF catheter ablation OR cryothermal ablation OR cryoballoon ablation OR cryoablation)  Indexes=SCI-EXPANDED, SSCI, A&HCI, CPCI-S, CPCI-SSH, BKCI-S, BKCI-SSH, ESCI, CCR-EXPANDED, IC Timespan=All years | 132,114 |
| #2 | ALL=(Same-day OR same-day discharge OR day-case)  Indexes=SCI-EXPANDED, SSCI, A&HCI, CPCI-S, CPCI-SSH, BKCI-S, BKCI-SSH, ESCI, CCR-EXPANDED, IC Timespan=All years | 21,437 |
| #3 | #1 AND #2  Indexes=SCI-EXPANDED, SSCI, A&HCI, CPCI-S, CPCI-SSH, BKCI-S, BKCI-SSH, ESCI, CCR-EXPANDED, IC Timespan=All years | 172 |

## The Cochrane library (August 21, 2021)

| **Search** | **Query** | **Results (No.)** |
| --- | --- | --- |
| #1 | (Atrial fibrillation* OR Catheter Ablation OR Auricular fibrillation* OR Transvenous Catheter Ablation* OR Electrical Catheter Ablation* OR Electric Catheter Ablation* OR Transvenous Electric Ablation* OR Transvenous Electrical Ablation* OR Radiofrequency Catheter Ablation* OR Percutaneous Catheter Ablation* OR atrial fibrillation catheter ablation* OR atrial fibrillation ablation* OR AF ablation* OR AF catheter ablation OR pulmonary vein isolation OR RF catheter ablation OR cryothermal ablation OR cryoballoon ablation OR cryoablation) | 15,220 |
| #2 | (Same-day OR same-day discharge OR day-case) | 4,031 |
| #3 | #1 AND #2 | 42 |

## Total results

| Data base | Results (No.) |
| --- | --- |
| PubMed | 129 |
| Embase | 481 |
| Scopus | 233 |
| Web of Science | 172 |
| The Cochrane library | 42 |
| Total | **1,057** |

**Supplementary Figures S1-S5**


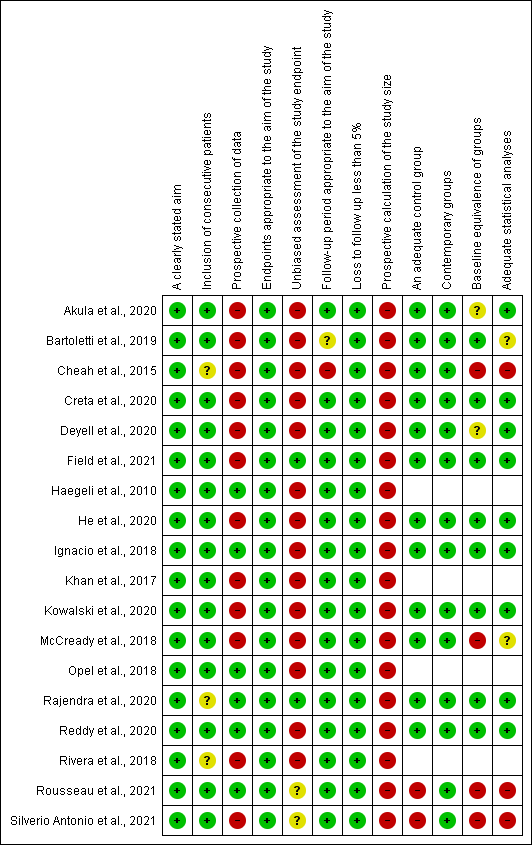


**Supplementary Figure S1.** Quality assessment of the included studies based on MINORS criteria


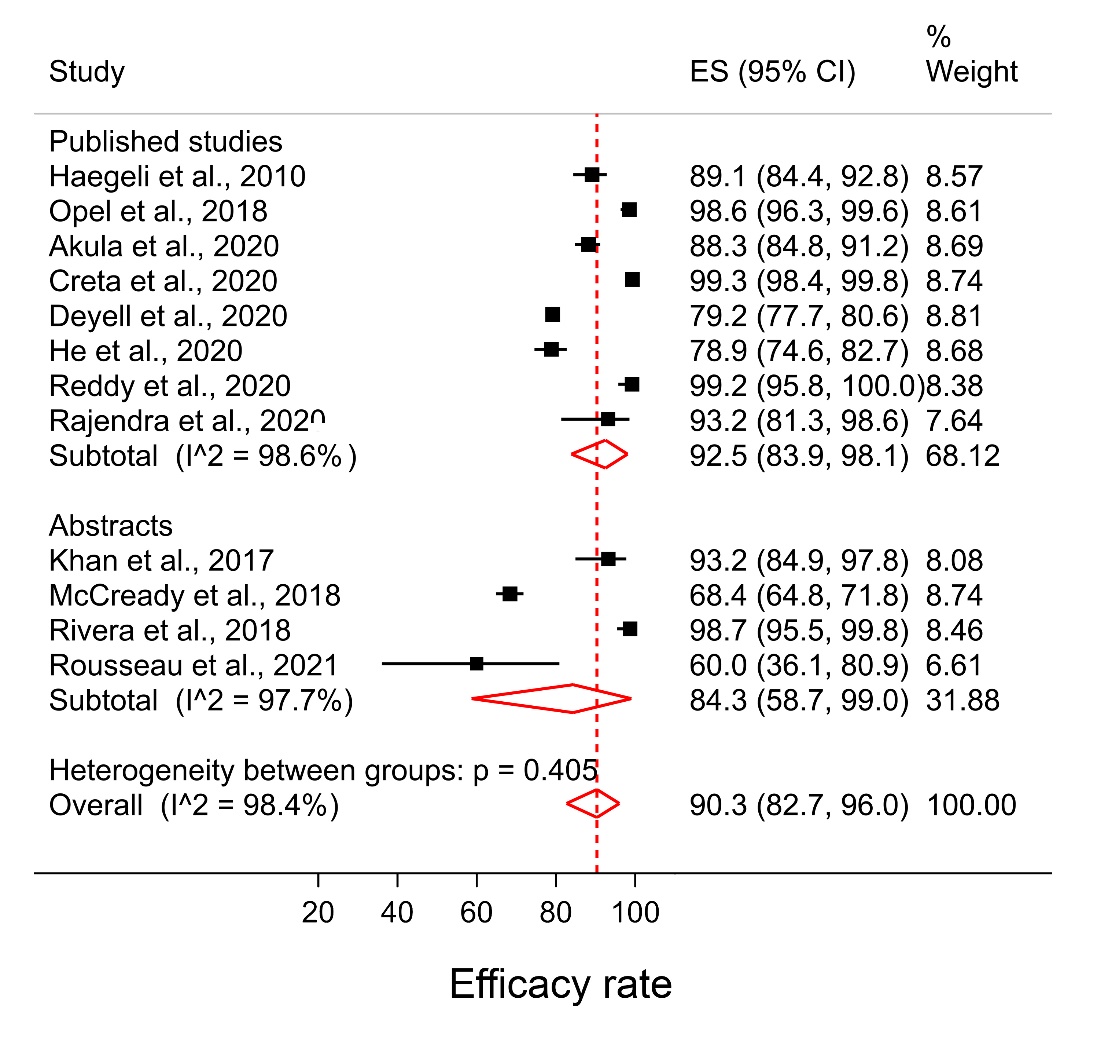


**Supplementary Figure S2.** Forest plots for efficacy rate in full texts and abstracts. ES, Effect Size (percentage); CI, Confidence Interval.


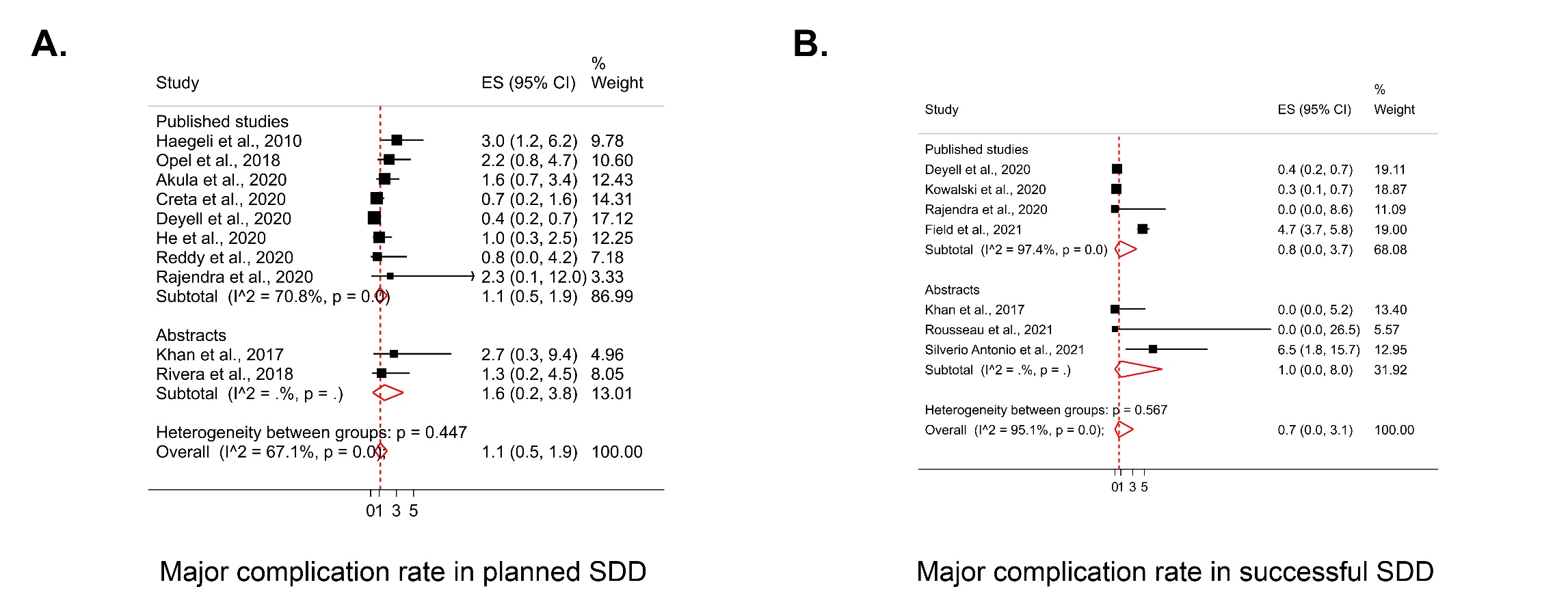


**Supplementary Figure S3.** Pooled proportion of major complications in A. planned same-day discharge (SDD) group, and B. Successful SDD group, in full texts and abstracts. ES, Effect Size (percentage); CI, Confidence Interval.


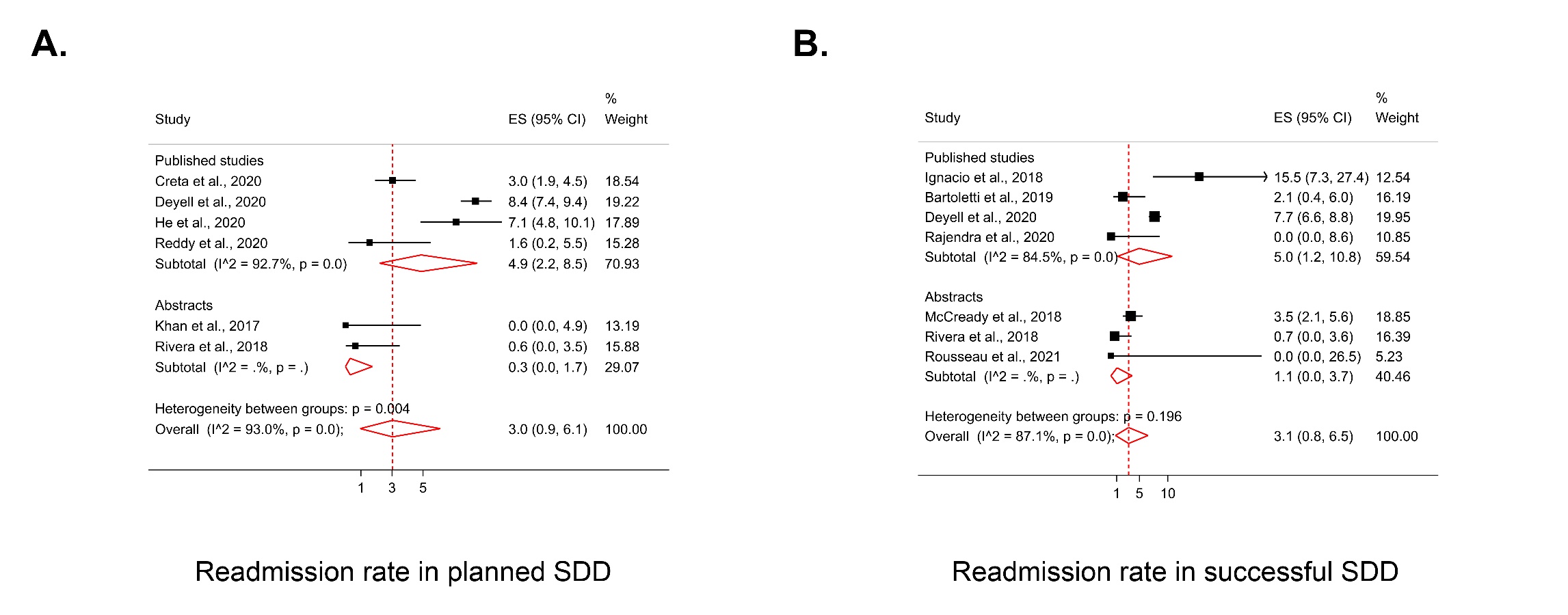


**Supplementary Figure S4.** Pooled proportion of early readmissions in A. planned same-day discharge (SDD) group, and B. Successful SDD group, in full texts and abstracts. ES, Effect Size (percentage); CI, Confidence Interval.

**
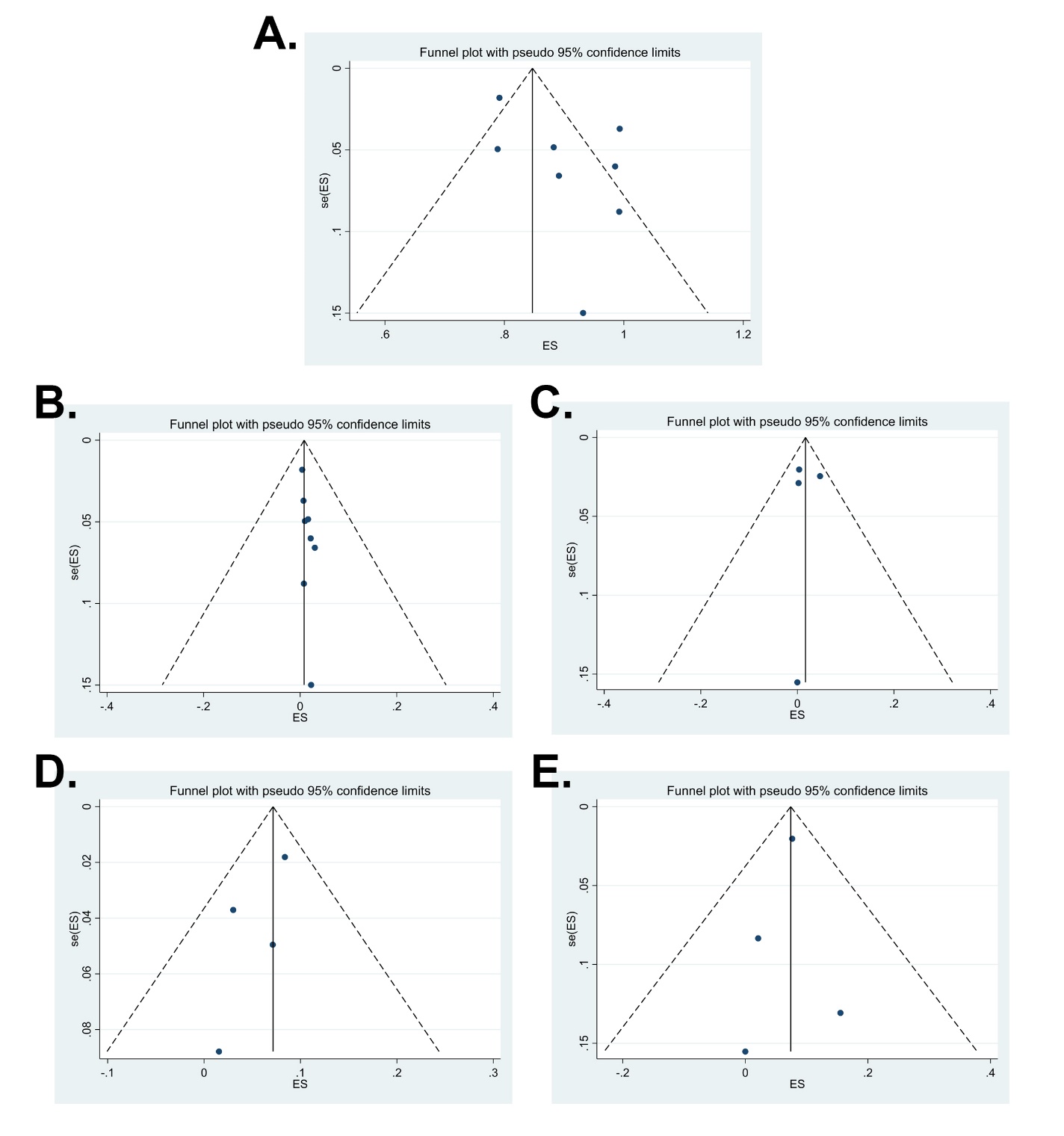
**

**Supplementary Figure S5.** Funnel plots for A. efficacy, B. major complications in planned SDD, C. major complications in successful SDD, D. readmission in planned SDD, and E. readmission in successful SDD. ES, Effect Size; SDD, Same-Day Discharge; SE, Standard Error.

**Supplementary Table S1** Characteristics of included abstracts

| Study, year | Country | Study design | Number of participants (SDD/ ON) | Mean age (years) | Male/ Female | Type of AF (paroxysmal/ persistent) | Patients with heart failure/ LVEF (%) | Body mass index (kg/m2) | CHA2DS2VASc score | Follow-up duration (months) | MINORS score |
| --- | --- | --- | --- | --- | --- | --- | --- | --- | --- | --- | --- |
| Cheah et al., 2015 | Australia | Retrospective, Single-center | 225 (72/153) | NR | NR | 225/0 | NR | NR | NR | 3 days | 11/24 |
| Khan et al., 2017 | USA | Retrospective, Single-center | 73 patients (69/4)  74 ablations | 62.3 ± NR | 54/20 | 49/24 | NR | NR | NR | 1 | 10/16 |
| McCready et al., 2018 | UK | Retrospective, Single-center | 936 (485/451) | NR | NR | NR | NR | NR | NR | 1 | 15/24 |
| Rivera et al., 2018 | Puerto Rico | Retrospective, Single-center | 157 (155/2) | 63.3 ± NR | 91/ 66 | NR | NR | NR | NR | 1 | 9/16 |
| Rousseau et al., 2021 | USA | Prospective, Single-center | 67  (12/55) | 63 ± NR | NR | NR | NR/  54.9 ± NR | 28.7 ± NR | 2.2 ± NR | 1 | 15/24 |
| Silverio Antonio et al., 2021 | Portugal | Retrospective, Single-center | 154  (62/92) | 61 ±  10.9 | 102/52 | 113/41 | NR/  NR | NR | NR | 6 | 13/24 |

Data are reported as number (percentage), mean ± standard deviation.

Abbreviations: AF, Atrial Fibrillation; LVEF, Left Ventricular Ejection Fraction; CFAE, Complex Fractionated Atrial Electrograms; MINORS, Methodological Index for Non-Randomized Studies; NR, Not Reported; ON, Overnight; SDD, Same-Day Discharge.

**Supplementary Table S2** Procedural and discharge characteristics

| Study, year | PVI ± additional ablation | Source of energy for ablation (No.) | Average procedure duration (minutes) | Anesthesia or  Sedation  No. (%) | Peri-procedural/  intra-procedural AC | Specific procedural, monitoring, and discharge characteristics of SDD groups |
| --- | --- | --- | --- | --- | --- | --- |
| Journal articles | |  |  |  |  |  |
| Haegeli et al., 2010 | PVI  ± additional linear lesions | Radiofrequency (230) | 201 ± 31 | Conscious sedation | Warfarin discontinuation 3 days prior to achieve INR < 2.0;  Resumption of oral AC on the day of ablation without bridging;  Intra-procedural heparin with an ACT target of 250-300s | Double transseptal puncture under fluoroscopy;  Radiofrequency energy delivered under the guide of Ensite NavX;  At least five hours of monitoring after the procedure;  No routine echocardiograms |
| Ignacio et al., 2018 | PVI | Radiofrequency (NR)  Cryoballoon (NR) | 91.5 ± NR | General anesthesia | NOACs were stopped 24 hrs. before; INR was targeted between 2-2.5;  Oral AC restarted 6 hrs. after the procedure;  Intra-procedural heparin with an ACT target of 350-450s | Transseptal puncture under fluoroscopy ± transesophageal or intracardiac echocardiogram; Esophageal temperature probe;  Phrenic nerve monitoring by diaphragmatic phrenic nerve pacing  Admission to the coronary care unit after the procedure and monitoring until discharge;  Phone call follow-up after 1 wk. |
| Opel et al., 2019 | PVI | Cryoballoon (276) | 63.5 ± 1.1 | Conscious sedation  (Patients requiring general anesthesia were excluded from SDD) | Uninterrupted warfarin or DOAC;  Intraprocedural weight-adjusted heparin without ACT monitoring | Phrenic nerve monitoring via pacing in the superior vena cava;  Protamine sulphate and Femostop to achieve hemostasis;  Transthoracic echocardiogram after the procedure;  Post-procedure monitoring for 4 hrs. and nurse-led discharge |
| Bartoletti et al., 2019 | PVI | Radiofrequency (522)  Cryoballoon (263) | S-SDD:  120 ± 29  ON:  153 ± 43 | Conscious sedation:  318(40.5%)  General anesthesia: 467(59.5%) | Uninterrupted warfarin; patients on NOACs were told to omit the morning dose; the usual dose was continued after ablation;  Intra-procedural heparin with an ACT target ≥ 300s | Use of compound motor action potentials to avoid phrenic nerve palsy; Post-procedure transthoracic echocardiogram if needed;  Protamine sulphate administer at the end of procedure;  4 hrs. of bed rest after ablation and then mobilization in the presence of nursing staff; any bleeding from access site mandated an extra 1 hr. of bedrest; provision with the number of a dedicated helpline upon discharge |
| Akula et al., 2020 | PVI ± linear lesions ± CFAE ablation | NR | NR | Monitored or general anesthesia | No standardized protocol for peri-procedural AC; Oral AC resumed on the day of ablation  Intra-procedural heparin with an ACT target between 300-350s | Transseptal puncture under fluoroscopy + intracardiac echocardiogram + pressure monitoring;  Post-procedure echocardiogram was not routine;  Monitoring in the cardiac unit for at least 6 hrs. post-ablation; reassessed by nurses before discharge |
| Creta et al., 2020 | PVI ± additional lesions | Radiofrequency (1341)  Cryoballoon (1287) | P-SDD:  79 ± 42  ON:  150 ± 83 | General anesthesia:  P-SDD: 61(8.4%)  ON:  661(34.8%) | Uninterrupted AC;  Intra-procedural heparin with an ACT target between 300-350s; | Mandatory vascular ultrasound before femoral puncture;  Transseptal puncture under fluoroscopy + pressure guidance ± transesophageal echocardiogram;  Protamine sulfate + Femostop or Z-sutures to achieve hemostasis;  Routine post-procedure transthoracic echocardiogram;  Monitoring in the elective ward for at least 4 hrs.;  Suitable patients were reassessed with a pre-specified workflow and discharged by the nurse |
| Deyell et al., 2020 | NR | Radiofrequency (2946)  Cryoballoon (108) | 190 (65-483) | General anesthesia:  3015(98.7%) | No specific peri-procedural AC protocol; Interrupted AC was resumed 6 hrs. after sheath removal;  Intra-procedural heparin was used | No routine post-procedure echocardiography;  Use of protamine sulphate was discretionary;  Monitoring in a cardiac short-stay unit with bedrest for up to 3 hrs.; bedrest duration extended if hemostasis was not achieved; Provision of phone access to clinic nurses |
| He et al., 2020 | PVI ± additional linear/focal ablations | Radiofrequency (603)  Cryoballoon (363) | 159.8 ± 76.4 | General anesthesia:  P-SDD: 7(2%)  ON: 31(6%) | Patients on warfarin had to have an INR between 2-3.5 on the day of ablation; Twice daily DOACs were taken 10 hrs. and daily DOACs were taken 20 hrs. before ablation;  Intraprocedural heparin to maintain ACT between 300-350s | Phrenic nerve monitoring via pacing in the superior vena cava;  Routine post-ablation transthoracic echocardiogram with repeat after 2-3 hrs.;  Protamine sulphate ± Z-sutures to achieve hemostasis;  Monitoring for 4 hrs. with 3 hrs. of bedrest |
| Kowalski et al., 2020 | PVI | Cryoballoon (2374) | NR | Conscious sedation or general anesthesia | Uninterrupted AC;  Intraprocedural heparin with ACT targeted at 300s | Transesophageal echocardiography before each case  Post-procedure echocardiography in all cases;  Protamine sulphate ± Z-sutures to achieve hemostasis;  Monitoring for 6 hours after procedure |
| Reddy et al., 2020 | PVI ± additional lesions ± CFAE ablation | Radiofrequency (315)  Cryoballoon (137) | S-SDD:  139.6 ± 37.8  ON:  160.7 ± 48.6 | General anesthesia:  S-SDD: 49(29.2%)  ON: 173(60.1%) | Warfarin continued uninterrupted; patients on NOACs omitted one dose pre-procedure;  Intra-procedural heparin with ACT targeted > 300s | Transseptal puncture under fluoroscopy;  Post-procedure echocardiogram was not routine;  Protamine sulphate to achieve hemostasis;  For cases with general anesthesia a recovery period was considered; monitoring for 3 hrs. with 2 hrs. of bedrest upon return to the ward |
| Rajendra et al., 2020 | PVI ±  Cavo-tricuspid isthmus | Radiofrequency (82) | S-SDD:  59 ± 15  ON:  67 ± 22 | General anesthesia | Uninterrupted AC | Patients identified for SDD had: stable anticoagulation, no bleeding history, no systolic heart failure, no pulmonary disease, no procedure within 60 days, body mass index < 35 kg/m^2^, acceptable CHA_2_DS_2_VASc score  Proximity to hospital, early procedure start.  Ablated with a porous tip contact force catheter  Six hours of bedrest following ablation, followed by intermittent ambulation  The study reports SDD eligibility and discharge criteria separately |
| Field et al., 2021 | NR | NR | NR | NR | NR | NR |
| Abstracts | |  |  |  |  |  |
| Cheah et al., 2015 | PVI | NR | NR | NR | NR | NR |
| Khan et al., 2017 | NR | Radiofrequency (30)  Cryoballoon (43) | NR | NR | NR | NR |
| McCready et al., 2018 | PVI | Radiofrequency (521)  Cryoballoon (413) | NR | NR | NR | NR |
| Rivera et al., 2018 | NR | Radiofrequency (NR)  Cryoballoon (NR) | NR | NR | NR | NR |
| Rousseau et al., 2021 | NR | NR | NR | NR | NR | The strategy for SDD was determined by cardiac arrhythmia allied health professionals. |
| Silverio Antonio et al., 2021 | PVI | Cryoballoon | NR | NR | NR | Only patients who underwent ablation before 4 PM were included in the analysis. |

Data are reported as number (percentage), mean ± standard deviation, or median (interquartile range) in case of non-normal distribution.

Abbreviations: AC, Anticoagulation; ACT, Activated Clotting Time; CFAE, Complex Fractionated Atrial Electrogram; INR, International Normalized Ratio; LA, Left Atrium; NOAC, Novel Oral Anticoagulant; NR, Not Reported; PVI, Pulmonary Vein Isolation; ON, Overnight; S-SDD, Successful Same-day Discharge; P-SDD, Planned Same-day Discharge.

**Supplementary Table S3** Sensitivity analyses

| Efficacy |  |  |  |  |
| --- | --- | --- | --- | --- |
| Study excluded | **Estimate %** | **LCI** | **UCI** | **I-squared** |
| Haegeli et al., 2010 | 93.0 | 83.3 | 98.8 | 98.80% |
| Opel et al., 2018 | 91.3 | 81.7 | 97.6 | 98.60% |
| Akula et al., 2020 | 93.1 | 83.0 | 99.0 | 98.80% |
| Creta et al., 2020 | 90.8 | 83.5 | 96.3 | 97.10% |
| Deyell et al., 2020 | 94.0 | 86.3 | 98.8 | 97.20% |
| He et al., 2020 | 94.0 | 84.6 | 99.3 | 98.70% |
| Reddy et al., 2020 | 91.1 | 81.5 | 97.5 | 98.70% |
| Rajendra et al., 2020 | 92.4 | 83.1 | 98.2 | 98.80% |
| Major complication – planned SDD | | | | |
| Study excluded | **Estimate %** | **LCI** | **UCI** | **I-squared** |
| Haegeli et al., 2010 | 0.8 | 0.3 | 1.5 | 62.10% |
| Opel et al., 2018 | 0.9 | 0.3 | 1.7 | 68.60% |
| Akula et al., 2020 | 1.0 | 0.4 | 1.9 | 70.60% |
| Creta et al., 2020 | 1.2 | 0.4 | 2.4 | 75.00% |
| Deyell et al., 2020 | 1.3 | 0.7 | 2.0 | 35.40% |
| He et al., 2020 | 1.1 | 0.4 | 2.1 | 74.50% |
| Reddy et al., 2020 | 1.1 | 0.4 | 2.0 | 74.80% |
| Rajendra et al., 2020 | 1.1 | 0.5 | 2.0 | 73.30% |
| Major complication – successful SDD | | | | |
| Study excluded | **Estimate %** | **LCI** | **UCI** | **I-squared** |
| Deyell et al., 2020 | 1.1 | 0.0 | 6.1 | 97.40% |
| Kowalski et al., 2020 | 1.2 | 0.0 | 6.0 | 97.90% |
| Rajendra et al., 2020 | 1.2 | 0.0 | 4.6 | 98.30% |
| Field et al., 2021 | 0.1 | 0.0 | 0.2 | 0.00% |
| Readmission – planned SDD |  |  |  |  |
| Study excluded | **Estimate %** | **LCI** | **UCI** | **I-squared** |
| Creta et al., 2020 | 5.9 | 3.1 | 9.4 | 83.80% |
| Deyell et al., 2020 | 3.8 | 1.4 | 7.2 | 83.30% |
| He et al., 2020 | 4.1 | 1 | 9.1 | 95.10% |
| Reddy et al., 2020 | 6 | 2.9 | 10.1 | 93.80% |
| Readmission – successful SDD |  |  |  |  |
| Study excluded | **Estimate %** | **LCI** | **UCI** | **I-squared** |
| Ignacio et al., 2018 | 3 | 0.1 | 8.7 | 86.40% |
| Bartoletti et al., 2019 | 6.3 | 1 | 14.8 | 81.70% |
| Deyell et al., 2020 | 3.9 | 0 | 14.5 | 85.90% |
| Rajendra et al., 2020 | 6.9 | 2.4 | 13.5 | 84.40% |

Abbreviations: SDD, same-day discharge; LCI, lower bound of confidence interval; UCI, upper bound of confidence interval.
